# Value of Information Analysis Applied to the Economic Evaluation of Interventions Aimed at Reducing Juvenile Delinquency: An Illustration

**DOI:** 10.1371/journal.pone.0131255

**Published:** 2015-07-06

**Authors:** Hester V. Eeren, Saskia J. Schawo, Ron H. J. Scholte, Jan J. V. Busschbach, Leona Hakkaart

**Affiliations:** 1 Institute for Medical Technology Assessment, Erasmus University Rotterdam, Rotterdam, the Netherlands; 2 Department of Psychiatry, section Medical Psychology and Psychotherapy, Erasmus Medical Center, Rotterdam, the Netherlands; 3 Viersprong Institute for Studies on Personality Disorders (VISPD), Halsteren, the Netherlands; 4 Behavioural Science Institute, Radboud University Nijmegen, Nijmegen, the Netherlands; National Institute for Public Health and the Environment, NETHERLANDS

## Abstract

**Objectives:**

To investigate whether a value of information analysis, commonly applied in health care evaluations, is feasible and meaningful in the field of crime prevention.

**Methods:**

Interventions aimed at reducing juvenile delinquency are increasingly being evaluated according to their cost-effectiveness. Results of cost-effectiveness models are subject to uncertainty in their cost and effect estimates. Further research can reduce that parameter uncertainty. The value of such further research can be estimated using a value of information analysis, as illustrated in the current study. We built upon an earlier published cost-effectiveness model that demonstrated the comparison of two interventions aimed at reducing juvenile delinquency. Outcomes were presented as costs per criminal activity free year.

**Results:**

At a societal willingness-to-pay of €71,700 per criminal activity free year, further research to eliminate parameter uncertainty was valued at €176 million. Therefore, in this illustrative analysis, the value of information analysis determined that society should be willing to spend a maximum of €176 million in reducing decision uncertainty in the cost-effectiveness of the two interventions. Moreover, the results suggest that reducing uncertainty in some specific model parameters might be more valuable than in others.

**Conclusions:**

Using a value of information framework to assess the value of conducting further research in the field of crime prevention proved to be feasible. The results were meaningful and can be interpreted according to health care evaluation studies. This analysis can be helpful in justifying additional research funds to further inform the reimbursement decision in regard to interventions for juvenile delinquents.

## Introduction

In order to guide policy decisions, it would be helpful to know the cost-effectiveness of interventions aimed at reducing juvenile delinquency. So far, cost-effectiveness analyses have informed an increasing number of reimbursement decisions in mental health-care [1,2]. Accordingly, the number of cost-effectiveness analyses in the field of crime prevention is increasing [2–10].

The inputs in a cost-effectiveness analysis can be uncertain, as available information about the costs and effects of interventions is rarely perfect. As a result, the decision whether or not to reimburse an intervention is marked by uncertainty. When a decision to reimburse an intervention turns out to be incorrect, it could lead to suboptimal interventions being approved. These interventions create costs in terms of foregone benefits and resources [11–15]. Further research may eliminate this uncertainty and optimize the reimbursement decision.

This study aims to estimate the added value of future cost-effectiveness research. This type of analysis is referred to as a ‘value of information’ analysis and was introduced as part of statistical decision theory [16,17]. It has already been applied in other research areas, such as engineering and environmental risk analysis [18], before being introduced into health technology assessment [11–15,19], where the application of this analysis is now widely adopted, as well as in the field of mental health care [20,21].

A value of information analysis reveals the value of conducting additional research and identifies the type of research that would be most useful. Its results can inform about further research on specific parameters, and more precisely inform the decision about which intervention should be reimbursed [22]. Furthermore, a value of information analysis can be used to prioritize future research, for example by highlighting the merits of certain types of research which might add to the reduction of the parameter uncertainty in cost-effectiveness analysis [15,23,24]. The potential value of further research could then be weighed against the costs of conducting this research in order to determine whether it is worthwhile (i.e. [11,12]).

Because a value of information analysis has not yet been applied in the field of crime prevention, we will present an example of this analysis based on an existing cost-effectiveness model in crime prevention and treatment [25]. We used two interventions aimed at reducing juvenile delinquency in the Netherlands, in adolescents aged 12–18 years. These interventions can be applied to prevent juvenile delinquency or used to prevent juveniles committing crimes in future, for example after an adolescent has been punished under the juvenile criminal laws. Juvenile law in the Netherlands applies to adolescents aged 12–17 years [26]. Not only the criminal act itself is important, but there is a strong focus on for example the background and moral development of the adolescent [26].

As the present study was set up as an illustration, data was used solely to demonstrate the method. We did not aim to test the superiority of one of the interventions that were used to illustrate the method. Therefore, this article merely presents a demonstration of the relevance of a value of information analysis in the field of crime prevention and treatment. The presented input data and results should be interpreted in this context. We will start with a short summary of an earlier illustrative cost-effectiveness analysis [25], and then introduce and illustrate the value of information analysis.

## Methods

### Interventions

We compared two interventions aimed at reducing juvenile delinquency. The ‘Kursushuis’ intervention (translated and referred to as the Course House) consists of a domestic foster home where several adolescents live for about 10 months and professional care is at close hand. The treatment costs and effects were described by Slot et al. [27]. The second intervention is a systemic intervention named Functional Family Therapy (FFT), which lasts about 4 to 6 months. The costs and effects of this intervention were obtained from a multicentre quasi-experimental study in the Netherlands [28]. The Medical Ethical Committee of the VU University Amsterdam approved this study (number 2008/152).

### Cost-effectiveness model

**Fig 1:**
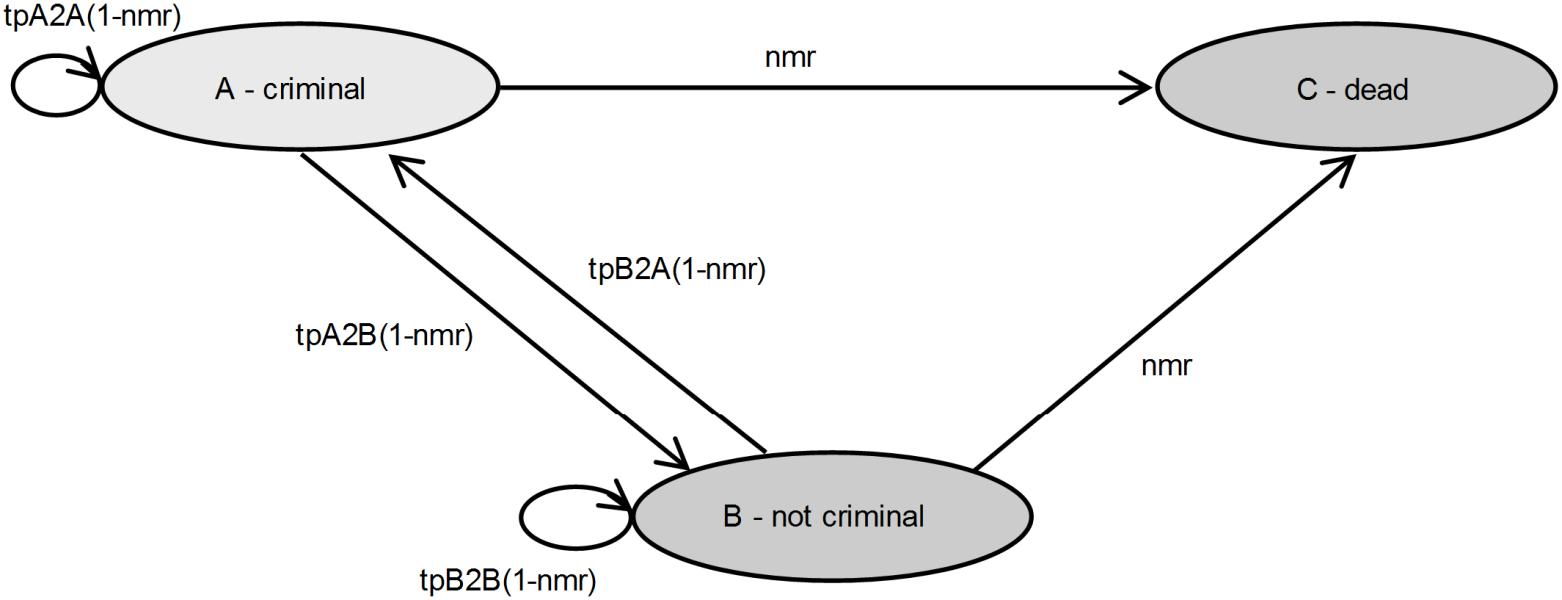
Markov model. nmr = natural mortality rate. tpA2A = transition probability of staying in state A. tpA2B = transition probability of moving from state A to state B. tpB2A = transition probability of moving from state B to state A. tpB2B = transition probability of staying in state B.

The Markov model that was used for the value of information analysis consists of three mutually exclusive model states: A) criminal behaviour, B) no criminal behaviour, and C) dead [25] (Fig 1). The time horizon of the model was 20 years, with a cycle length of six months [25]. A societal perspective was taken and results were expressed as costs per Criminal Activity Free Year (CAFY) [25].

In line with health economic guidelines [29], the input parameters in the model were threefold. The first group of parameters were the transition probabilities. These reflect the probability that an adolescent transitions through the states. The measure of time an adolescent spends in a non-criminal state is used to estimate a CAFY. Criminal activity was based on the adolescents’ self-reported contact with the police in connection with he/she having committed one or several crimes; having had no contacts was defined as criminal-activity free and having had one or more contacts as criminally active. Transition probabilities were extrapolated until the age of 30, as we integrated parts of the long-term stabilising effects described by Moffitt [25,30]. Dying because of committing crimes was not reflected in the CAFY. Adolescents were assumed to face a risk of death equivalent to the age specific mortality rates in the general population [31]. The second group consisted of costs of health-care use, productivity losses, and other societal costs such as costs of the criminal justice system. Both costs outside health care, and health care costs were included, such as the costs of visiting a psychiatrist or psychologist. As the family system is involved in the interventions provided, we included both the costs of the adolescent and those of one of the parents. The model state costs were fixed over time until the adolescent was 23 years. It was assumed that from that age onwards not all cost categories (such as a family guardian or foster care) would remain relevant. The third group comprised the intervention costs. The costs of one completed FFT treatment was calculated to be approximately €10,900 per adolescent, whereas the Course House was about €37,800 (retrieved from Slot et al. [27]). Both costs were extrapolated to 2013 Euro’s accounting for inflation based on the consumer price index [32]. The cost and effects in the model were discounted (i.e.[33,34]), according to the guidelines for economic evaluations in the Netherlands [29].

To represent the uncertainty of each model parameter, we assigned parameter distributions (S1 Table). In a probabilistic analysis, uncertainty was simulated by running the model 10,000 times using a cohort of subjects and each time taking different parameter estimates from the parameter distributions [11,12]. These 10,000 unique sets of parameter values were used to estimate the mean expected cost-effectiveness. For further details on the cost-effectiveness model, we refer to Schawo et al. [25].

### Cost-effectiveness analysis

**Fig 2:**
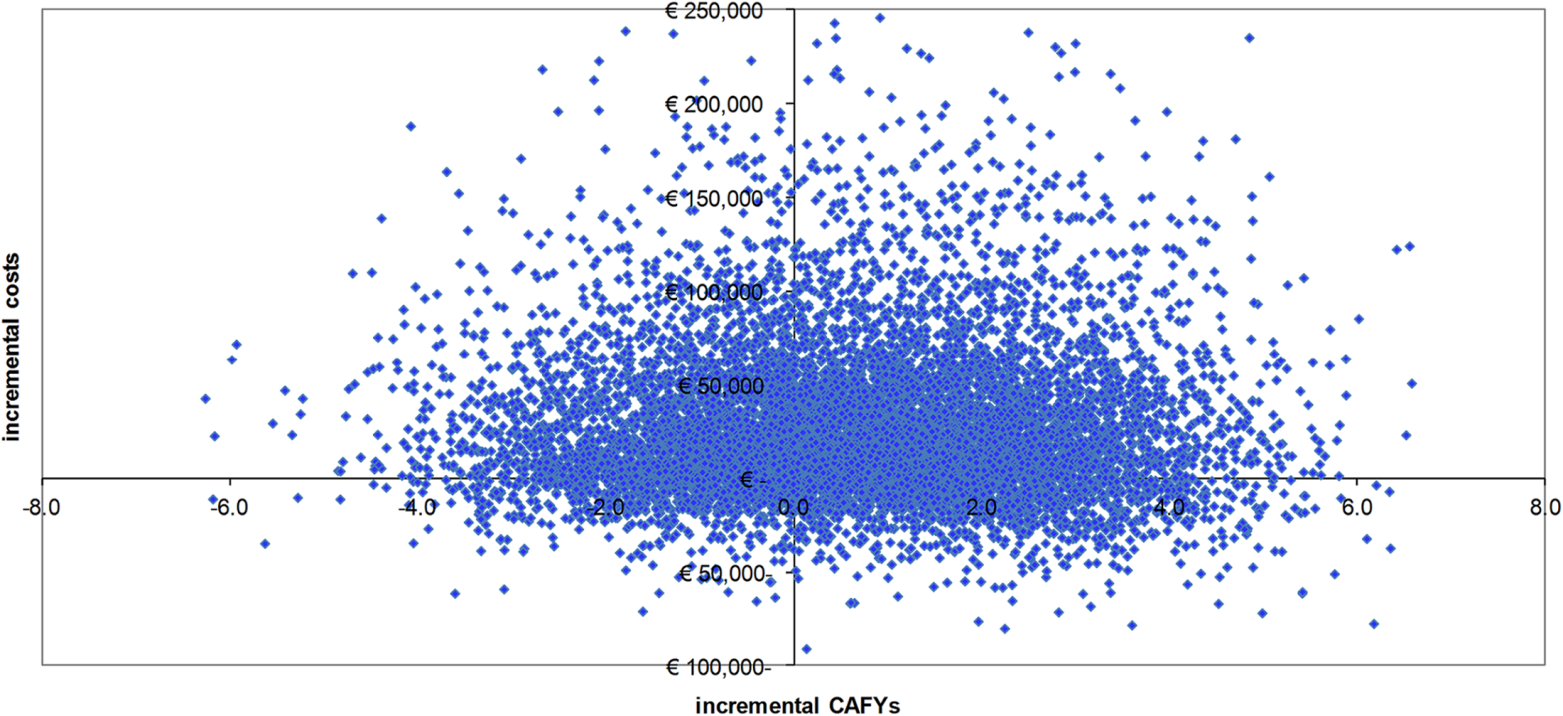
Incremental cost-effectiveness plane for Course House compared with FFT (10,000 simulations). FFT = Functional Family Therapy CAFY = Criminal Activity Free Year.

The stochastic model resulted in the relative cost-effectiveness outcomes of the Course House intervention compared with FFT, represented as incremental costs/CAFY (Table 1; Fig 2). It showed that the Course House was more effective than FFT, but also produced higher costs. The cumulative number of CAFYs for the Course House exceeded the number of CAFYs for FFT by 0.7, while the incremental costs of the Course House exceeded those of FFT by €26,800, thereby positioning the intervention in the North East quadrant of the cost-effectiveness plane [35] (Fig 2). The incremental cost-effectiveness ratio (ICER) of the Course House compared with FFT was 39,000 €/CAFY.

**Table 1:** Cost-effectiveness results over 20 years^a^.

Intervention	Cost	CAFY	ICER^b^	NMB^c^
Course House	€249,000	12.4	€39,000	€641,200
FFT	€222,200	11.7	-	€618,700

CAFY, criminal activity free year; ICER, incremental cost-effectiveness ratio; NMB, net monetary benefit; FFT, Functional Family Therapy.

^a^ The results represented were averaged over the 10,000 simulations run.

^b^ The ICER was calculated as the difference in cost divided by the difference in CAFYs between the Course House and FFT.

^c^ The NMB was calculated by multiplying CAFYs by the WTPvalue of €71,700 per CAFY and subtracting cost. The Course House is cost-effective compared with FFT, because the NMB of the Course House is higher than the NMB of FFT. Due to decimals, the numbers in the table multiplied do not give the exact NMB values represented in this table.

### Parameter uncertainty

The influence of parameter uncertainty on the model outcomes was shown in the cost-effectiveness acceptability frontier (CEAF). In the CEAF, the probability of being cost-effective compared to the other intervention is shown for the intervention with the highest expected net monetary benefit (NMB) for a range of societal willingness-to-pay (WTP) values per CAFY, and is therefore cost-effective compared with the alternative, given a certain WTP [36,37]. Here, the overall maximum expected net benefit guides the decision on which intervention is cost-effective compared with the alternative intervention [36,37]. The NMB was calculated by multiplying CAFYs by the WTP value per CAFY and subtracting cost [11,12]. The CEAF is illustrated in the results’ section.

### Value of information analysis

In the value of information analysis, the parameter uncertainty in the model is monetarized. More precisely, we estimated the value of ‘knowing everything’: the ‘expected value of having perfect information’ (EVPI) [12,14]. Having perfect information would eliminate parameter uncertainty and optimize the reimbursement decision. In estimating the ‘value of knowing everything’, the EVPI places an upper boundary on the value of performing further research [11,12]. It can be interpreted as the maximum value society ‘should’ be willing to pay for additional evidence to reduce decision uncertainty around which intervention is preferred and, therefore, inform the reimbursement decision in the future [11,12]. The EVPI is computed by first taking the difference between the expected NMB with perfect information and the expected NMB with current information per simulation. This difference is equal to the expected benefits foregone when making the decision based on current evidence [11,12]. Comparing the EVPI estimates with the costs of this future research reveals whether further research is worthwhile.

As the value of further information is related to the size of the eligible population of adolescents to be treated, the EVPI was multiplied with the eligible population of adolescents in the population EVPI (pEVPI). About 825 adolescents annually were assumed to be eligible for FFT in the Netherlands. When we discount this number over five years, which is the assumed lifetime of the intervention for which additional research would be useful [11,29], it resulted in an eligible population of 3,820 adolescents. We assumed that the eligible number of adolescents for the Course House was equal to that for FFT.

In a value of information analysis one could also focus on specific groups of model parameters. To identify the model parameters that contribute to most of the uncertainty and for which future research is the most promising, we estimated the expected value of partial perfect information (EVPPI) [11,12]. The EVPPI was estimated using the Sheffield Accelerated Value of Information application of Strong et al. [38]. Multiplying the EVPPI values with the eligible population results in the population EVPPI (pEVPPI).

The EVPI and EVPPI not only depend on the uncertainty of the model parameters, but also on the WTP per CAFY. In the absence of a WTP per CAFY in the Netherlands, we used WTP estimates to reduce crime of Cohen et al. [39,40]. These WTP values per crime indicate the value society wants to pay to prevent one crime, for example €32,200 per burglary (Table 2). Table 2 provides an overview of these estimates, adjusted for inflation and purchasing power parities [41]. Although WTP to prevent one crime is definitely not equal to WTP per CAFY, we used it to illustrate what is meant by WTP in crime prevention and how the concept can be used in a value of information analysis. We hereby implicitly assumed that one crime is committed per year, and thus exactly one crime per year is avoided in a CAFY. We estimated the EVPI and EVPPI for various WTP values, and we chose an average WTP value to illustrate the result in the results section, which was €71,700 (Table 2).

**Table 2:** Willingness-to-pay values for crimes (Cohen & Piquero, 2009).

Crime	WTP in 2007 dollars	WTP in 2013 euro’s
Murder	$140,000	€128,700
Rape	$290,000	€266,600
Armed robbery	$280,000	€257,400
Robbery	$39,000	€35,900
Aggravated assaults	$85,000	€78,100
Simple assaults	$19,000	€17,4500
Burglary	$35,000	€32,200
Moter vehicle theft	$17,000	€15,600
Larceny	$4,000	€3,700
Druk driving crash	$60,000	€55,200
Arson	$115,000	€105,700
Vandalism	$2,000	€1,800
Fraud	$5,500	€5,100
Other offenses	$1,000	€900
**Average**	**$140,000**	**€71,700**

WTP, willingness-to-pay

The model parameters were grouped into the following ten subsets to indicate the direction of research as a result of the EVPPI analysis: research on 1) transition probabilities for FFT; 2) transition probabilities for the Course House; 3) direct health-care costs of the criminal state; 4) direct health-care costs of the non criminal state; 5) direct non health-care costs related to the criminal state; 6) direct non health-care costs related to the non criminal state; 7) indirect non health-care costs related to the criminal state; 8) indirect non health-care costs related to the non criminal state; 9) intervention costs of FFT; and 10) intervention costs of the Course House.

## Results

### Model uncertainty

**Fig 3:**
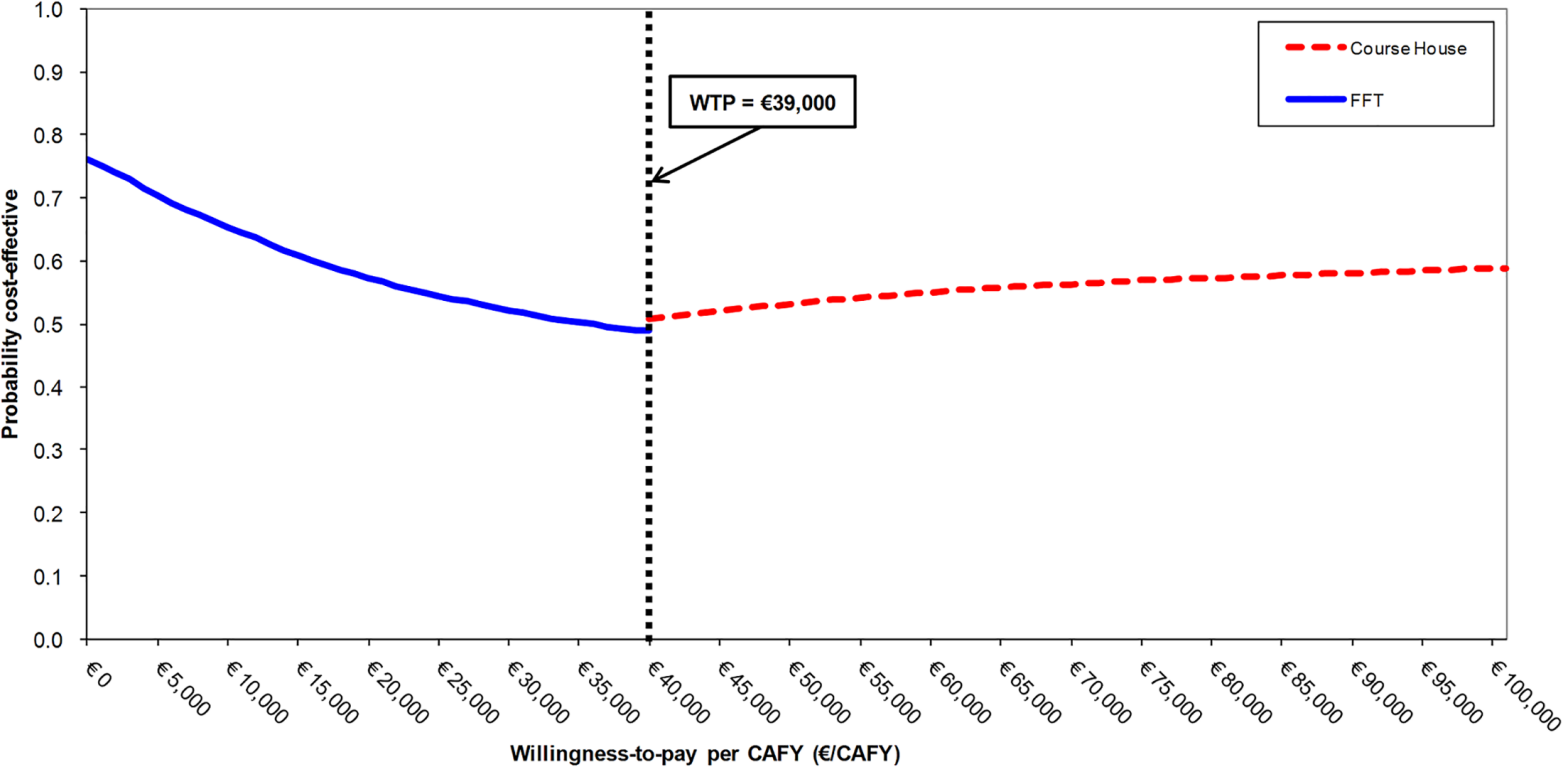
Cost-effectiveness Acceptability Frontier (CEAF). FFT = Functional Family Therapy. CAFY = Ciminal Activity Free Year. WTP = Willingness-to-pay.

The CEAF shows that FFT had the highest NMB for a WTP ranging from €0–€39,000 (Fig 3). At a WTP of €39,000, FFT was cost-effective in 49% of the 10,000 model simulations, or a probability of 0.49, whereas the Course House was cost-effective in 51% of the simulations. Above the €39,000 WTP, the Course House had the highest NMB and thus was the optimal intervention. This switching point in the CEAF is where the NMB for FFT is equal to the NMB of the Course House. At this point, the WTP was exactly equal to the ICER value (€39,000 per CAFY).

The CEAF (Fig 3) shows a large error probability. At the WTP of €71,700 the Course House was cost-effective in 57% of the 10,000 model simulations, which suggests that there is an error probability of 0.43 that could be reduced by collecting additional evidence.

### Value of information analysis

In order to know the value of reducing the error probability and to assign a value to additional research, we estimated the EVPI. Table 3 illustrates the EVPI estimation (based on Soeteman et al. [21]). The table shows the generated NMB for each intervention for 6 of the 10,000 simulations, given a WTP value of €71,700 per CAFY. The EVPI was determined as follows: First, we assumed that decision makers have perfect information for each simulation instead of making one single choice over all simulations. For example, for simulation 1 and 2, this would result in the choice for FFT (see Table 3). Second, we determined the choice based on current information. In this case, the Course House had the highest expected NMB (€641,200) over all simulations and hence was the preferred intervention. Finally, we took the difference between the decision based on perfect information per simulation and the optimal choice over all simulations. This difference resulted in the EVPI value or the benefits foregone per simulation. The expectation of all benefits foregone over the 10,000 simulations is the EVPI per adolescent, which is €46,000 at a WTP value of €71,700 per CAFY. Perfect information for an individual adolescent was thus valued at €46,000. Multiplying this EVPI value by 3,820 eligible adolescents resulted in a pEVPI of €176 million. This pEVPI value suggests that, at a societal WTP value of €71,700 per CAFY, there is room to reduce the parameter uncertainty in the model by a maximum of €176 million.

**Table 3:** Calculation of expected value of perfect information (EVPI) for individual adolescent.

Simulation		Net monetary benefits^a^		Maximum net benefit	Benefits foregone
		Course House	FFT		
	*Expectation*	***€641*,*200***	*€618*,*700*	*€687*,*200*	*€46*,*000*
1		€481,000	**€650,000**	€650,000	€169,000
2		€553,800	**€710,300**	€710,300	€156,500
3		€513,800	**€768,000**	€768,000	€254,200
4		**€717,500**	€562,700	€717,500	€ 0
5		€516,200	**€671,000**	€671,000	€154,800
…	…	…	…	…	…
10,000		**€602,300**	€587,200	€602,300	€0

^a^ Net monetary benefit (NMB) was calculated by multiplying CAFYs by the WTP value of €71,700 per CAFY and subtracting cost.

Explanation:

Decision based on current information: Course House.

Decision based on perfect information: bold.

Expected net benefit with current information: €641,200.

Expected net benefit with perfect information: €687,200.

Expected value of perfect information (EVPI): €687,200−€641,200 = €46,000.

**Fig 4:**
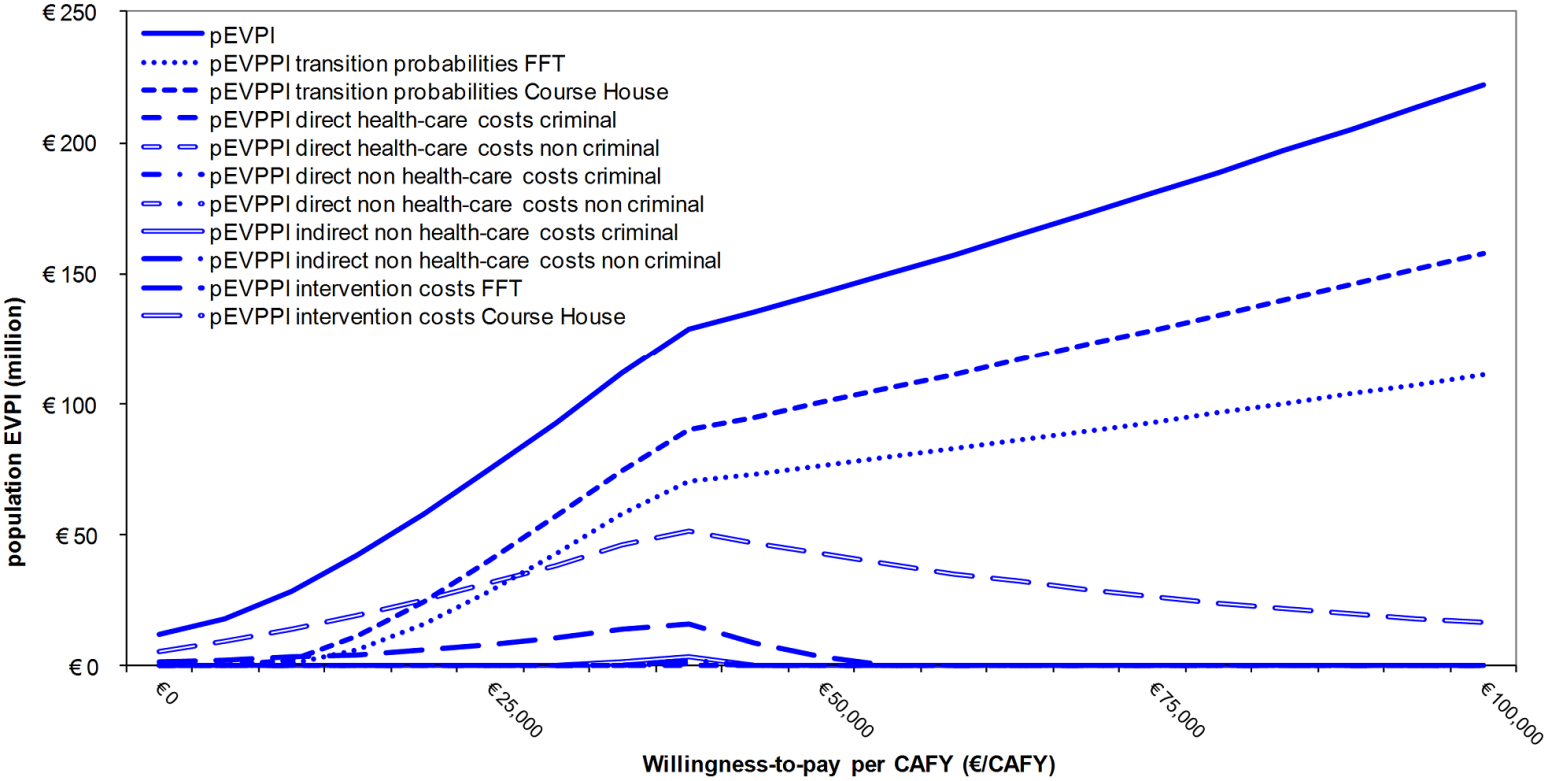
population Expected Value of Perfect Information (pEVPI) and population Expected Value of Partial Perfect Information (pEVPPI) curve. FFT = Functional Family Therapy. CAFY = Criminal Activity Free Year. pEVPI = population Expected Value of Perfect Information. pEVPPI = population Expected Value of Partial Perfect Information.

Perfect information can be valued at different WTP values. The extent of the monetarized uncertainty surrounding the decision for a range of WTP values is represented in the pEVPI curve. Fig 4 presents the pEVPI curve for an eligible population of 3,820 adolescents. As an example we consider the point where research costs society €50 million (i.e. the pEVPI value at the y-axis in Fig 4). At this point further research would potentially be cost-effective if society were willing to pay more than €17,600 per CAFY (i.e. the value at the x-axis, if the pEVPI is €50 million). At lower values of the WTP per CAFY, the benefits of further research cannot offset the costs [11,42]. At a WTP of €39,000 per CAFY, the pEVPI shows a local maximum of €127 million. At this point, the parameter uncertainty in the model is the highest and thus decision uncertainty is highest, as already shown in the CEAF curve (Fig 3).

Perfect information of subsets of parameters was valued in the pEVPPI. This pEVPPI was estimated for a range of WTP values (Fig 4). At the illustrative WTP value of €71,700 per CAFY, future research would be most valuable for three subsets of parameters: the transition probabilities and the intervention costs of the Course House and the transition probabilities of FFT (see Fig 4). The pEVPPI of the transition probabilities of the Course House was €125 million (€32,700 per adolescent), and the pEVPPI for the transition probabilities of FFT was €91 million (€23,800 per adolescent). The pEVPPI for the intervention costs of the Course House was €28 million (€7,400 per adolescent). The pEVPPIs for the direct non health-care costs of the criminal state and the non criminal state were respectively €8,400 and €43,300 (respectively €2 and €11per adolescent). The pEVPPIs for the other parameter groups were all estimated to be zero (Fig 4), meaning there was no potential value of further research into these parameters. Given a WTP of €71,700 per CAFY further research for these parameters would not reduce decision uncertainty. The EVPI and EVPPI values depend highly on the WTP value per CAFY, as can be seen in Fig 4 and S2 Table. At a WTP of for example €40,000, there was indeed potential value of further research into all model states costs. Note that due to the interactions within the model structure, the pEVPPI for the groups of parameters do not sum up to the overall pEVPI for the model (see Fig 4) [11,42].

## Discussion

While cost-effectiveness analyses are increasingly being used in the field of crime prevention, the value of further research has not yet been estimated for comparison between interventions aimed at reducing juvenile delinquency. An earlier developed cost-effectiveness model was used to estimate this value of further research. This study demonstrated that it was feasible to estimate the value of conducting further research in this context, using a value of information framework common in health economic evaluations. The results can be interpreted as similar to cost/QALY (quality-adjusted life year) studies in health care evaluation.

In this value of information analysis, the results indicated the parameters for which further research was valuable. Our findings show particular uncertainty in three groups of parameters: the transition probabilities of the Course House and of FFT, and to a lesser extent, the intervention costs of the Course House and the direct non health-care costs in both model states. Performing additional research in the suggested fields can reduce parameter uncertainty, and hence, can reduce decision uncertainty.

Therefore, the results of a value of information analysis can prioritize further research to optimize the final reimbursement decision, thereby increasing the probability that adolescents will be assigned to the intervention that is cost-effective, compared with the alternative. Given this information, future interventions could be reimbursed (or not), and they could also be approved ‘only in research’ (OIR) (i.e. further research is required before the intervention can be approved) or ‘approved with research’ (AWR) (i.e. research can be conducted while the intervention is approved) [43,44]. For example, from this study we can conclude that given a WTP of €40,000 per CAFY, the Course House could be ‘approved with research’. The Course House would then be reimbursed while further research would be required, for example on the effectiveness of the Course House. Current practice in adolescent care in the Netherlands illustrates this approval condition: the Dutch Youth Institute identifies effective youth interventions, while still conducting research on the effectiveness of some of these interventions [45]. However, approval might lead to irrecoverable costs when the approval is revised due to subsequent research revealing that the Course House was not as effective as expected. Then, approval ‘only in research’ might be preferred, because commitment to future costs is avoided until the results of further research are known. Approval might even be dependent on any change in the effective price of an intervention [44,46].

This study was a first attempt to apply a value of information framework to the field of crime prevention and treatment of juvenile delinquents. Therefore, some considerations should be kept in mind. The value of information analysis estimates the monetary value of eliminating all or part of the parameter uncertainty of the presented model. However, two other sources of uncertainty can influence the results: structural and methodological uncertainty [47,48]. Structural uncertainty relates to structural aspects of the model [47,49,50], such as the conceptual framework or the transitions between the model states [50], and it can lead to different estimated model outcomes (i.e.[51]). This structural uncertainty is likely to be present in our model. For example, we did not account for the severity of crimes in the model states or the elevated risk of death for adolescents in the criminal state (i.e. [52,53]). The uncertainty of these aspects was not represented in the current value of information analysis. Future cost-effectiveness models in the field of crime prevention should therefore carefully characterize the structural uncertainty [50], and account for it when possible, for example by parameterization [49,50] or model averaging [47,50,54]. The second additional source of uncertainty is methodological uncertainty, which relates to the analytical method chosen [48,49]. Our model also represents some methodological uncertainties, such as whether or not to include the costs of crime in the model (i.e. [55]). Here, three methodological uncertainties in our model are discussed in more detail. These uncertainties could be resolved through, for example, formulating guidelines (i.e.[47,49]) to model cost-effectiveness research in the field of crime prevention.

The first methodological uncertainty concerns the societal perspective used in the model, which means that we included the costs and effects relevant to society. When considering this perspective in health care, the focus is merely on the patient, whereas this will be different in the area of crime prevention and treatment (i.e.[56]). In this study, we already included the direct and indirect costs of one parent, as well as direct non medical costs of the adolescent, such as the costs of contact with the police. Other costs that we did not account for, but are nevertheless relevant in the field of crime prevention are: the effect of the intervention reflected in both costs and effects, such as increased wellbeing and reduced productivity losses (i.e.[56]), in regard to family members (e.g. parents or siblings of the adolescents). Further additional categories are reduced victim costs and increased victim wellbeing, the reduction of the number of out-of-home placements, the reduction of the costs of committed crimes to society, reduced costs of avoided crimes to society and the value of reduced fear of crime (i.e. [39,56]).

The second methodological uncertainty deals with the WTP value for a CAFY. Although we used the WTP values of Cohen et al. [39,40] to illustrate the use of WTP in crime prevention, WTP to prevent a crime like burglary is definitely not equal to WTP per CAFY. Therefore, it is important to carefully estimate the WTP value per CAFY. In this study, for example, we could have weighted the WTP values of Cohen et al. [39,40] by the frequency of the crimes as yearly committed by the adolescents in this study, or by the number of yearly registered crimes in the Netherlands. For clarity reasons and due to a lack of more detailed data regarding the crimes committed, we chose not to use a weighted WTP value. Furthermore, for the WTP values used, it is not exactly known which components of crime, such as investigation, prosecution, witnesses, legal aid, prevention programs, the costs to victims, and the valuation of fear [39] are included in this valuation [39,57]. Therefore, further research is needed into which categories of costs of crime are included in a WTP value, before determining what society is willing to pay for one CAFY. The cost categories included in the cost-effectiveness model should be reflected in the WTP value and vice versa. Also, other estimations of the societal WTP might be considered. These could, for example, be based on the cost of crime using a bottom-up approach or a breaking-down approach [58]. These methods take into account only the costs of crime, not the willingness to reduce crime levels.

Third, to estimate a WTP per CAFY, it should be known what type of criminal activity is avoided in a CAFY. The seriousness of the crime, the number of times the crime is committed and the types of criminal activity can also be taken into account in defining criminal activity. Furthermore, it is important to decide on how to measure criminal activity. The CAFY used in our study was based on the adolescents’ self-reported contact with the police. However, criminal activity may as well be determined on the basis of police registries [27], contacts with other judicial institutions [6–8], rates of reconviction [3], or a delinquency score [5]. Different definitions of criminal activity can influence the model results. For example not all committed crimes are recorded in police registrations, while self-reported measures could yield socially desirable answers. Using the CAFY in further research thus requires a clear definition of criminal activity.

A final remark on this analysis concerns the interventions chosen. FFT and the Course House were chosen to illustrate the analysis in the field of crime prevention. The interventions under study, however, could be replaced by other interventions aimed at reducing juvenile delinquency, such as Multisystemic Therapy, Multidimensional Foster Treatment Care or Multidimensional Family Therapy [45]. Contrary to a broader range of cost-effectiveness studies in the UK and US (i.e. [4,59]), in the Netherlands, to the best of our knowledge, the cost-effectiveness of such interventions has not yet been investigated, except for a cost-benefit analyses of ‘Maatregel Inrichting Stelstelmatige Daders’ or a case study into ‘Strafrechtelijke Opvang Verslaafden’ [56,60], which are both aimed at adults. Furthermore, in studying interventions in this field, the context of the interventions under study is highly important. In our illustration, we assumed that in practice, the interventions would be applied completely equivalently. However, preferences for an intervention may influence the choice for a certain intervention in reality, such as earlier experience with an intervention, specific characteristics of an adolescent, or the availability of the intervention itself. In our illustration, FFT may be, for example, used more often to avoid committing crimes, whereas the Course House could be used as an addition to a punishment under juvenile justice law, where the adolescent has already committed a crime. There may then be a higher probability of recidivism if the adolescents already have a history of committing crimes [61]. These non-equivalent baseline situations may influence the measured effectiveness of the intervention. Moreover, the situation after treatment may also be different. This may affect the acceptance of possible or required further care (i.e.[61]), and therefore may influence the final degree of committing crimes in the future. In modelling the cost-effectiveness of interventions in the field of crime prevention, the application of interventions in practice should therefore be taken into account in a cost-effectiveness model, or at least, this should be clarified when modelling the cost-effectiveness of such interventions.

In conclusion, an analysis to estimate the value of performing further research had not yet been conducted in the field of crime prevention. The findings of the current study illustrate how such an analysis might be estimated and interpreted in this field. Future investment in cost-effectiveness research on interventions aimed at reducing juvenile delinquency could use this value of information framework to efficiently conduct further cost-effectiveness research.

## Supporting Information

S1 Table Model parameters and parameter distributions. (PDF)

S2 Table pEVPI and pEVPPI for a range of WTP values. (PDF)
