# Supplementary material for: Value of Information Analysis Applied to the Economic Evaluation of Interventions Aimed at Reducing Juvenile Delinquency: An Illustration
Source: PLoS One. 2015 Jul 6;10(7):e0131255. doi: 10.1371/journal.pone.0131255 (PMC4493049; doi:10.1371/journal.pone.0131255)
Supplement: S1 Table — (PDF) [file pone.0131255.s001.pdf]

Table S1. Model parameters and parameter distributions

EVPPI group of parameters	Parameters						
	**Transition probabilities**	**Probability**	**Events**	**Complements**		**Distribution**	**Reference**
transition probabilities FFT	***FFT***						
	tpA2A	0.23	6	20		Dirichlet	Multicentre trial
	tpA2B	0.77	20	6		Dirichlet	Multicentre trial
	tpB2A	0.37	7	12		Dirichlet	Multicentre trial
	tpB2B	0.63	12	7		Dirichlet	Multicentre trial
transition probabilities Course House	***Course House***						
	tpA2A	0.39	13	20		Dirichlet	Slot et al. (1992)
	tpA2B	0.61	20	13		Dirichlet	Slot et al. (1992)
	tpB2A	0.24	4	13		Dirichlet	Slot et al. (1992)
	tpB2B	0.76	13	4		Dirichlet	Slot et al. (1992)
	**Intervention costs**	**Mean**	**SE mean***	**alpha**	**beta**	**Distribution**	**Reference**
intervention costs FFT	FFT	€ 10,900	€ 10,900	1	10900	Gamma	Costdata mental health institutions
intervention costs Course House	Course House	€ 37,800	€ 37,800	1	37800	Gamma	Slot et al. (1992)
			** SE was not known, therefore taken conservative and set equal to mean*				
	**Model state costs**	**Mean**	**SE mean**	**alpha**	**beta**	**Distribution**	**Reference**
	Criminal state (A)						
direct health-care costs criminal	Direct health-care - adolescent						
	Psychiatrist	€ 112	€ 67	2.8	39.9	Gamma	Multicentre trial
	Psychologist	€ 183	€ 103	3.2	57.5	Gamma	Multicentre trial
	Psychiatric nurse	€ 47	€ 47	1.0	47.2	Gamma	Multicentre trial
	Social worker	€ 113	€ 78	2.1	54.2	Gamma	Multicentre trial
	GP	€ 43	€ 26	2.7	15.6	Gamma	Multicentre trial
	GP at school	€ 3	€ 3	1.0	3.0	Gamma	Multicentre trial

	Pediatrician	€ 0	€ 0	0.0	0.0	Gamma	Multicentre trial
	Medical specialist	€ 8	€ 1	100.0	0.1	Gamma	Multicentre trial
	Alternative healer	€ 0	€ 0	0.0	0.0	Gamma	Multicentre trial
	Family guardian	€ 0	€ 0	0.0	0.0	Gamma	Multicentre trial
	Youth welfare agency	€ 142	€ 75	3.6	39.5	Gamma	Multicentre trial
	ER	€ 66	€ 50	1.7	38.4	Gamma	Multicentre trial
	Foster care	€ 0	€ 0	0.0	0.0	Gamma	Multicentre trial
	Residential institution	€ 25	€ 25	1.0	25.3	Gamma	Multicentre trial
	Day hospitalization	€ 0	€ 0	0.0	0.0	Gamma	Multicentre trial
	Hospitalization	€ 1,438	€ 1,438	1.0	1437.5	Gamma	Multicentre trial
	Centre for addiction treatment	€ 0	€ 0	0.0	0.0	Gamma	Multicentre trial
	Direct health-care - parent						
	Psychiatrist	€ 61	€ 61	1.0	61.1	Gamma	Multicentre trial
	Psychologist	€ 55	€ 47	1.4	40.5	Gamma	Multicentre trial
	Psychiatric nurse	€ 0	€ 0	0.0	0.0	Gamma	Multicentre trial
	Social worker	€ 6	€ 6	1.0	5.9	Gamma	Multicentre trial
	GP	€ 94	€ 71	1.7	53.9	Gamma	Multicentre trial
	Medical officer	€ 12	€ 7	3.3	3.7	Gamma	Multicentre trial
	Medical specialist	€ 21	€ 15	2.0	10.9	Gamma	Multicentre trial
	Alternative healer	€ 9	€ 9	1.0	9.1	Gamma	Multicentre trial
	Family guardian	€ 0	€ 0	0.0	0.0	Gamma	Multicentre trial
	Centre for addiction treatment	€ 80	€ 80	1.0	79.7	Gamma	Multicentre trial
direct non health-care costs criminal	Direct non health-care - adolescent						
	Council of child protection	€ 25	€ 16	2.3	10.7	Gamma	Multicentre trial
	Bureau Halt	€ 44	€ 27	2.8	15.9	Gamma	Multicentre trial
	Police	€ 90	€ 37	6.0	15.0	Gamma	Multicentre trial

	Lawyer	€ 57	€ 57	1.0	57.1	Gamma	Multicentre trial
	Court	€ 29	€ 29	1.0	28.6	Gamma	Multicentre trial
	Social rehabilitation	€ 171	€ 111	2.4	71.4	Gamma	Multicentre trial
	Incarceration costs	€ 0	€ 0	0.0	0.0	Gamma	Multicentre trial
indirect non health-care costs criminal	Indirect non health-care - adolescent						
	Time spent on exercises as part of intervention	€ 3	€ 2	1.9	1.6	Gamma	Multicentre trial
	Indirect non health-care - parent						
	Absence from work	€ 1,317	€ 835	2.5	528.8	Gamma	Multicentre trial
	Inefficiency at work	€ 4,450	€ 2,775	2.6	1730.4	Gamma	Multicentre trial
	Productivity losses due to unpaid support	€ 0	€ 0	0.0	0.0	Gamma	Multicentre trial
	Productivity losses due to paid support	€ 0	€ 0	0.0	0.0	Gamma	Multicentre trial
	Time spent on exercises as part of intervention	€ 2	€ 2	1.0	2.1	Gamma	Multicentre trial
	Non Criminal state (B)						
direct health-care costs non criminal	Direct health-care - adolescent						
	Psychiatrist	€ 31	€ 17	3.3	9.2	Gamma	Multicentre trial
	Psychologist	€ 78	€ 45	3.1	25.4	Gamma	Multicentre trial
	Psychiatric nurse	€ 0	€ 0	0.0	0.0	Gamma	Multicentre trial
	Social worker	€ 60	€ 45	1.8	33.9	Gamma	Multicentre trial
	GP	€ 42	€ 15	8.3	5.1	Gamma	Multicentre trial
	GP at school	€ 0	€ 0	0.0	0.0	Gamma	Multicentre trial
	Pediatrician	€ 0	€ 0	0.0	0.0	Gamma	Multicentre trial
	Medical specialist	€ 12	€ 8	2.0	5.6	Gamma	Multicentre trial
	Alternative healer	€ 0	€ 0	0.0	0.0	Gamma	Multicentre trial
	Family guardian	€ 16	€ 12	1.9	8.4	Gamma	Multicentre trial
	Youth welfare agency	€ 47	€ 27	3.0	15.7	Gamma	Multicentre trial

	ER	€ 30	€ 2	289.6	0.1	Gamma	Multicentre trial
	Foster care	€ 0	€ 0	0.0	0.0	Gamma	Multicentre trial
	Residential institution	€ 247	€ 247	1.0	246.6	Gamma	Multicentre trial
	Day hospitalization	€ 0	€ 0	0.0	0.0	Gamma	Multicentre trial
	Hospitalization	€ 9	€ 9	1.0	9.4	Gamma	Multicentre trial
	Centre for addiction treatment	€ 0	€ 0	0.0	0.0	Gamma	Multicentre trial
	Direct health-care - parent						
	Psychiatrist	€ 0	€ 0	0.0	0.0	Gamma	Multicentre trial
	Psychologist	€ 80	€ 40	3.9	20.3	Gamma	Multicentre trial
	Psychiatric nurse	€ 0	€ 0	0.0	0.0	Gamma	Multicentre trial
	Social worker	€ 17	€ 17	1.0	16.8	Gamma	Multicentre trial
	GP	€ 18	€ 7	5.9	3.0	Gamma	Multicentre trial
	Medical officer	€ 6	€ 6	1.0	6.1	Gamma	Multicentre trial
	Medical specialist	€ 39	€ 2	292.4	0.1	Gamma	Multicentre trial
	Alternative healer	€ 0	€ 0	0.0	0.0	Gamma	Multicentre trial
	Family guardian	€ 27	€ 19	1.9	14.3	Gamma	Multicentre trial
	Centre for addiction treatment	€ 0	€ 0	0.0	0.0	Gamma	Multicentre trial
direct non health-care costs non criminal	Direct non health-care - adolescent						
	Council of child protection	€ 0	€ 0	0.0	0.0	Gamma	Multicentre trial
	Bureau Halt	€ 12	€ 9	1.6	7.2	Gamma	Multicentre trial
	Police	€ 25	€ 15	3.0	8.4	Gamma	Multicentre trial
	Lawyer	€ 0	€ 0	0.0	0.0	Gamma	Multicentre trial
	Court	€ 0	€ 0	0.0	0.0	Gamma	Multicentre trial
	Social rehabilitation	€ 36	€ 29	1.6	23.0	Gamma	Multicentre trial
	Incarceration costs	€ 0	€ 0	0.0	0.0	Gamma	Multicentre trial
indirect non health-	Indirect non health-care - adolescent						

care costs non criminal	*Time spent on exercises as part of intervention*	€ 4	€ 4	1.2	3.4	Gamma	Multicentre trial
	Indirect non health-care – parent						
	Absence from work	€ 1,020	€ 558	3.3	305.9	Gamma	Multicentre trial
	Inefficiency at work	€ 3,866	€ 1,503	6.6	584.5	Gamma	Multicentre trial
	Productivity losses due to unpaid support	€ 0	€ 0	0.0	0.0	Gamma	Multicentre trial
	Productivity losses due to paid support	€ 0	€ 0	0.0	0.0	Gamma	Multicentre trial
	Time spent on exercises as part of intervention	€ 8	€ 5	2.7	2.8	Gamma	Multicentre trial

Italics - costs not relevant from 23 years onwards

EVPPI, Expected Value of Partial Perfect Information; FFT, Functional Family Therapy; SE, Standard error
