# Supplementary material for: Value of Information Analysis Applied to the Economic Evaluation of Interventions Aimed at Reducing Juvenile Delinquency: An Illustration
Source: PLoS One. 2015 Jul 6;10(7):e0131255. doi: 10.1371/journal.pone.0131255 (PMC4493049; doi:10.1371/journal.pone.0131255)
Supplement: S2 Table — (PDF) [file pone.0131255.s002.pdf]

Table S2. pEVPI and pEVPPI for a range of WTP values

WTP	transition probabilities FFT	transition probabilities Course House	direct health-care costs criminal	direct health-care costs non criminal	direct non health-care costs criminal	direct non health-care costs non criminal	indirect non health-care costs criminal	indirect non health-care costs non criminal	intervention costs FFT	intervention costs Course House	pEVPI
**€ 0**	€ 0	€ 0	€ 0	€ 0	€ 0	€ 0	€ 0	€ 0	€ 1,798,900	€ 5,646,700	€ 11,911,500
**€ 5,000**	€ 23,000	€ 0	€ 0	€ 0	€ 1,700	€ 0	€ 2,300	€ 0	€ 2,462,300	€ 9,568,600	€ 18,197,300
**€ 10,000**	€ 919,600	€ 1,990,500	€ 0	€ 0	€ 12,400	€ 0	€ 13,300	€ 0	€ 3,351,400	€ 14,051,900	€ 28,674,800
**€ 15,000**	€ 6,402,400	€ 11,155,100	€ 0	€ 0	€ 21,200	€ 900	€ 41,300	€ 0	€ 4,519,500	€ 19,242,800	€ 42,195,800
**€ 20,000**	€ 16,263,300	€ 24,720,800	€ 600	€ 0	€ 29,800	€ 0	€ 107,000	€ 0	€ 6,029,400	€ 25,103,700	€ 57,847,600
**€ 25,000**	€ 28,832,200	€ 40,322,400	€ 8,900	€ 0	€ 34,300	€ 0	€ 255,400	€ 0	€ 7,980,200	€ 31,601,900	€ 74,913,300
**€ 30,000**	€ 42,919,000	€ 57,149,700	€ 16,900	€ 0	€ 60,400	€ 0	€ 609,000	€ 10,700	€ 10,567,700	€ 38,703,200	€ 92,970,400
**€ 35,000**	€ 57,863,500	€ 74,630,700	€ 41,300	€ 0	€ 264,800	€ 10,700	€ 1,761,100	€ 559,100	€ 14,004,000	€ 46,337,600	€ 111,815,800
**€ 40,000**	€ 70,749,600	€ 89,965,700	€ 590,500	€ 357,800	€ 1,546,500	€ 1,089,900	€ 3,732,300	€ 2,326,300	€ 15,926,700	€ 51,773,800	€ 128,621,700
**€ 45,000**	€ 73,470,900	€ 95,078,000	€ 0	€ 0	€ 185,800	€ 24,000	€ 374,800	€ 21,300	€ 8,788,000	€ 47,140,300	€ 135,339,200
**€ 50,000**	€ 76,407,100	€ 100,394,600	€ 0	€ 0	€ 75,900	€ 32,500	€ 20,300	€ 0	€ 3,432,200	€ 42,879,500	€ 142,393,000
**€ 55,000**	€ 79,537,400	€ 105,903,400	€ 0	€ 0	€ 42,900	€ 35,000	€ 800	€ 0	€ 427,800	€ 38,985,800	€ 149,713,600
**€ 60,000**	€ 82,789,400	€ 111,483,800	€ 0	€ 0	€ 25,100	€ 37,500	€ 0	€ 0	€ 0	€ 35,421,100	€ 157,238,100
**€ 65,000**	€ 86,144,500	€ 117,144,600	€ 0	€ 0	€ 14,200	€ 40,000	€ 0	€ 0	€ 0	€ 32,177,300	€ 164,940,500
**€ 70,000**	€ 89,582,300	€ 122,808,500	€ 0	€ 0	€ 9,400	€ 42,500	€ 0	€ 0	€ 0	€ 29,245,900	€ 172,780,800
**€ 75,000**	€ 93,083,900	€ 128,578,700	€ 0	€ 0	€ 7,300	€ 44,900	€ 0	€ 0	€ 0	€ 26,603,300	€ 180,738,000
**€ 80,000**	€ 96,638,800	€ 134,392,600	€ 0	€ 0	€ 6,700	€ 47,400	€ 0	€ 0	€ 0	€ 24,164,400	€ 188,804,300
**€ 85,000**	€ 100,232,100	€ 140,405,200	€ 0	€ 0	€ 6,100	€ 49,900	€ 0	€ 0	€ 0	€ 21,956,200	€ 196,955,500
**€ 90,000**	€ 103,864,300	€ 146,115,500	€ 0	€ 0	€ 5,400	€ 52,300	€ 0	€ 0	€ 0	€ 19,947,500	€ 205,186,900
**€ 95,000**	€ 107,525,900	€ 152,098,000	€ 0	€ 0	€ 4,800	€ 54,700	€ 0	€ 0	€ 0	€ 18,102,800	€ 213,485,700
**€ 100,000**	€ 111,211,000	€ 157,921,800	€ 0	€ 0	€ 4,100	€ 57,200	€ 0	€ 0	€ 0	€ 16,420,600	€ 221,852,400

WTP, willingness-to-pay; FFT, Functional Family Therapy; pEVPI, population Expected Value of Perfect Information; pEVPPI, population Expected Value of Partial Perfect Information
